# Family-level intelligence and maternal health: A cross-cohort, cross-generational longitudinal study using the NLSY

**DOI:** 10.1016/j.intell.2025.101966

**Published:** 2025-10-24

**Authors:** Linda Wänström, Patrick O’’Keefe, Graciela Muniz-Terrera, Stacey Voll, Frank D. Mann, Sean Clouston, Scott Hofer, Joseph L. Rodgers

**Affiliations:** a Linköping University, Department of Computer and Information Science, Sweden; b Oregon Health and Sciences University, Department of Neurology, USA; c Ohio University, Heritage College of Osteopathic Medicine, USA; d University of Victoria, Department of Psychology, Institute of Lifelong Health and Aging, USA; e Stony Brook University, Department of Medicine, USA; f Vandelbilt University, Psychological Sciences, USA; g Pentara Corporation, USA

**Keywords:** Family iq, Child intelligence, Maternal health, NLSY, Multilevel growth curve models, Canonical correlation

## Abstract

This study examines the association between family-level intelligence metrics, and maternal health outcomes in middle age, as captured in the National Longitudinal Survey of Youth. Building on past research documenting links between maternal intelligence and health, our study expands the inquiry by exploring how both variations and trends in family-level intelligence are associated with maternal middle-age health. We use multilevel modeling analysis to extract family intelligence levels and growth scores from children’s Peabody Individual Achievement Test of math, reading recognition and reading comprehension. We use two time-points, ten years apart, to extract levels and growth scores from maternal middle-aged health data. We then use canonical correlation analysis to examine the associations between family intelligence and maternal health. Our results show a positive association between family cognition and maternal health. Families with greater math and reading recognition levels experience better levels of maternal health outcomes. Patterns also suggest that low levels in math and reading comprehension are related to larger declines in physical health. We discuss implications of intellectual development in the family, noting that higher family intelligence not only holds intrinsic value but also is associated with improved maternal health outcomes. We discuss a possible “Flynn effect transfer” within the family context, where intellectual advancement correlates with positive health trajectories in midlife mothers. Future research could extend these insights to explore further downstream effects on both maternal and child well-being.

## 1. Introduction

This paper investigates the relationship between family intelligence and maternal health. We define family-level intelligence and growth as functions of the intelligence levels and growths of children in the household, and maternal health as functions of mental and physical levels and growth in middle-aged mothers. Our study expands upon previous research on relationships between intelligence and health by focusing on family-level intelligence (intelligence of the children and the mother) and by examining cross-generational links (mothers and children) between both levels and changes in family intelligence and maternal health.

### 1.1. The intelligence-health relationship, background

The relationship between intelligence and health outcomes has been a focal point of numerous studies. Gottfredson (2004) proposed intelligence as the critical “missing link” in understanding health disparities. Across the literature, the correlation between intelligence, whether assessed during childhood or later in life, and health outcomes, encompassing both physical and mental health, is predominantly positive (e.g., Hardie & Landale, 2013). Wraw et al. (2015) found positive associations between Armed Forces Qualification Test (AFQT) scores in adolescence and middle-aged health outcomes in the National Longitudinal Survey of Youth (NLSY) data. A meta-analysis (Calvin et al., 2011) revealed a consistent negative association between childhood intelligence and all-cause mortality (also see Martin et al., 2004, and Leon et al., 2009, among others), and Calvin et al. (2017) later linked childhood intelligence to ultimate mortality patterns. The relationship between intelligence and health outcomes is an important part of cognitive epidemiology (Deary et al., 2021), an emerging and developing discipline, and Deary et al. suggested that attention to specific and focused health outcomes comprises much of this research arena. In our study, we view health outcomes more broadly, focusing both on a physical health index from a general health battery and on depression scores.

Research has also found associations between mothers’ health and their children’s intelligence. Maternal depressive symptoms have been linked to reduced cognitive performance in children and increased behavioral issues (Caplan et al., 1989; Soares et al., 2024; Sutherland et al., 2021; Turney, 2012). Pre-pregnancy body mass index (BMI) has been negatively associated with children’s cognitive performance, albeit modestly (Basatemur et al., 2013). Additionally, children’s developmental delays have been connected to both adverse maternal mental (Baker et al., 1997) and physical health statuses (Eisenhower et al., 2009).

Given the links between mothers’ health outcomes and their children’s cognitive performance, which may go in either direction or be bidirectional in a causal sense, we view the family entity (comprised of e.g. the children or of the children and the parents in the household) as an important entity to examine when studying relationships between maternal health and intelligence.

### 1.2. Family intelligence

The family entity has been the focus in some previous research when studying family intelligence. O’Keefe and Rodgers (2017) examined trends in children’s PIAT-math scores in the NLSY (National Longitudinal Survey of Youth) longitudinal data and found that between-family variance dominated within-family variance, suggesting that the family unit was the most important explanatory source. Wänström et al. (2023) used a multilevel modeling approach to estimate growth curves for the children’s PIAT-math, reading recognition, and reading comprehension scores in the NLSY data and found differential family patterns for children in different family cohorts, where family cohort was defined with reference to either the maternal cohort (i.e. the mother’s birth year), or with reference to the beginning of the family unit (i.e. the year the first child was born). These results indicated that the Flynn effect (cohort increases in intelligence scores), which had mostly been investigated at the individual level, operated also at the family level. We review the Flynn effect literature in the next section.

### 1.3. Increases in intelligence scores – Individual and family Flynn effects

Flynn’s landmark 1984 study (Flynn, 1984) sparked widespread interest in the temporal dynamics of intelligence, revealing an average increase of approximately three IQ points per decade throughout the 20th century. Since then, research on the Flynn effect has diversified into empirical documentation of global intelligence increases, theoretical explorations of its causes, methodological advancements, and integrative analyses (Rodgers, 2023) and meta analyses (Pietschnig & Voracek, 2015; Trahan et al., 2014; Wongupparaj et al., 2023). Although the vast majority of research investigated the effect at the individual level, some research has looked more closely at the role of the family. Rodgers (2014) showed that the Flynn effect can “stand in” for within-family birth order effects when researchers use cross-sectional data (i. e., the patterns are Flynn effects, but mis-interpreted in cross-sectional data to be birth order effects). Several more recent papers have suggested that the Flynn effect emerges primarily (but not solely) at the family level. As reviewed in the previous section, O’Keefe and Rodgers (2017) found important between-family variance in PIAT-scores, which suggested that the family unit was a primary source from which the Flynn effect emerged. Wänström et al. (2023) found family Flynn effects for both levels and growths in family PIAT-scores. The sizes of those family Flynn effects were substantially larger, for the “start of the family”-cohort measure, than the usual individual Flynn effects identified in many of the past studies.

Theories have emerged that try to explain the family Flynn effects. Rodgers and O’Keefe (2023) developed a new “synthetic theory”, the Parental Executive Model, that emphasizes the role that parents play in creating the Flynn effect. This theory suggests that many parents actively draw on the processes captured in previous Flynn effect theories (e.g., health improvements, educational innovations, nutritional developments, technology, etc.) to facilitate intellectual development in their children. In addition, a cross-generational process occurs, whereby children becoming more intelligent over time become better at facilitating their own children’s intellectual development when they become parents. Other theoretical developments overlap in their content and predictions and also emphasize the family as the potential locus for intellectual growth. In particular, the Life History perspective (e.g., Dunkel et al., 2021; Woodley, 2012) has been shown to be among the most effective explanations for the Flynn effect. Pietschnig and Voracek (2015) created a competition among a number of theories to explain relevant features of the Flynn effect, and the Life History perspective was declared the most effective.

These family Flynn effect findings and theories motivate both empirical and theoretical attention to family-level research, especially when it comes to studying intelligence. This, together with the positive relationships between intelligence and health (including in particular mothers’ health and their children’s cognitive performance) provides motivation for our study, which focuses on relationships between family intelligence and maternal health outcomes. Our study’s contribution to the literature linking intelligence and health outcomes is twofold. First, we operationalize intelligence at the family level. Second, we examine these links cross-generationally between children and maternal measures, and we study how both levels and changes underlie these links. We view our study as correlational, as we do not assume any causal direction between maternal health and family intelligence. However, establishing these links could enhance our understanding of the underlying mechanisms linking family intelligence and health in future studies.

## 2. Materials and methods

Wänström et al. (2023) used multilevel models of children’s PIAT-scores, in the NLSY data, to examine Family Flynn effects. These models predicted PIAT-scores at both individual and family levels and produced within-child, between-child-within-family, and between-family variance. Family Flynn effects were estimated using family cohort measures (mother’s birth year or first child’s birth year) in these models. In the current study we use residuals from these multilevel models of the children’s PIAT-scores (without the cohort measures). Extracting family residual scores will enable us to study relationships between family intelligence and health, as described in a later section.

### 2.1. Sample

The National Longitudinal Survey of Youth 1979 (NLSY79), managed by the Bureau of Labor Statistics, is a longitudinal survey of 12,686 US adolescents and young adults from 8770 households, initially aged 14 to 21 at the end of 1978. The NLSY Children (NLSYC) includes all biological children of the NLSY79 mothers, totaling 11,504 individuals. Children from small sub-samples of NLSY79 mothers, who were omitted in the 1980s for budgetary reasons, are excluded from the NLSYC. NLSYC participants have been biennially surveyed since 1986, establishing intricate familial connections (which are documented in Rodgers et al., 2016). These children (51 % male, 49 % female; 53 % non-black/non-Hispanic, 28 % Black, 19 % Hispanic or Latino) underwent biennial evaluations using the Peabody Individual Achievement Test (PIAT) in mathematics, reading recognition, and reading comprehension from ages 5 to 15, between 1986 and 2014, yielding repeated measures at two-year intervals. Each child eligible by age underwent up to five cognitive assessments, offering a comprehensive view of family and child development. The average age at assessment was 9.75 years, and respondents were born between 1970 and 2009, predominantly between 1982 and 1991. By 2010, ages ranged from 1 to 39. Distribution of cognitive assessments across families varied: 25.7 % had one child assessed, 39.9 % had two, 22.0 % had three, 8.4 % had four, 2.7 % had five, and a small fraction had six to ten children evaluated. Our study analyzes these family units longitudinally, tracking the development of children born to NLSY79 mothers through the NLSY79 and NLSYC databases.

### 2.2. Measures

We used the Peabody Individual Achievement Test (PIAT) metrics, specifically PIAT-math, PIAT-reading recognition, and PIAT-reading comprehension, for our analyses. The dataset included 11,530 children from the NLSYC cohort, with the following specifics. 9233 children of 4055 mothers were assessed repeatedly, up to five times, by the PIAT math test, resulting in 34,498 math assessments. The corresponding specifics were 34,358 assessments from 9220 children of 4051 mothers on the reading recognition test, and 33,655 assessments from 9199 children of 4046 mothers on the reading comprehension test. As in Wänström et al. (2023), we used raw scores, rather than normalized values, to examine developmental progression across different ages. We note that these tests are strictly achievement tests, which are usually viewed as separate from intelligence tests. More recently, this separation has been questioned, as a number of achievement tests correlate as highly with IQ tests as IQ tests do with one another. For example, Frey and Detterman (2004) showed that the SAT correlates 0.82 with a measure of g in the NLSY, and suggest that “the SAT is mainly a test of g” (p. 373). We use the PIAT scores as measures of cognitive functioning within the relevant domains, as in previous NLSY research using PIAT scores (e.g., Ang et al., 2010; Rodgers & Wänström, 2007).

The PIAT-math subtest comprises 84 progressively challenging items. Each age group began the test with a different starting item. If a child missed initial items, they reverted to the start point of the next younger age bracket. A ‘basal’ level was established when a child correctly answered five consecutive items, from which point they proceeded until they incorrectly answered five out of seven items. The final PIAT-math score reflects the number of the last correctly answered item minus the count of errors post-basal.

The PIAT-reading recognition subtest evaluates a child’s ability to silently read and articulate words, featuring 84 items ranging from simple (e.g. “run”, “play”, “jump”) to complex (e.g. “credulity”, “disaccharide”, “apothegm”). The PIAT-reading comprehension test assesses understanding through 66 items, where a child reads a sentence and selects a matching picture. Scoring for the reading subtests mirrors the PIAT-math structure, with final scores adjusted based on the sequential correct responses and errors beyond the basal level. However, children with a reading recognition score under 19 were not assessed for reading comprehension, equating their scores in both areas.

In the NLSY79, health assessments were conducted for mothers as they reached ages 40 and 50, starting during the survey years of 1998 and 2008, respectively. These assessments included the Short-Form 12-question (SF-12) and the 7-item Center for Epidemiological Studies Depression Scale (CES-D). The SF-12 is a comprehensive measure of self-reported mental and physical health, while the CES-D is specifically designed to assess the prevalence of depressive symptoms and is considered a more objective measure of mental health compared to the SF-12 mental health subscale. To explore the relationship between family growth curves, as indicated by the children’s PIAT scores, and the mothers’ health in midlife, we utilized data from the 7-item CES-D and the physical health component of the SF-12. Health data were available for 3420 mothers at age 40 and for 3199 mothers at age 50.

The NLSY data do not include longitudinal maternal IQ scores like the ones we have for children, but we do have a single maternal measure collected in 1980, when respondents were ages 15–23, the Armed Forces Qualifying Test (AFQT). We note that, although the AFQT was developed as an achievement test, it has often been used in past research as a proxy for an IQ test, as we use it in the current study. Alternative measures for maternal intelligence do not exist in the data, apart from some cognitive functioning measures in health assessments, which are not fully applicable to our research objective.

### 2.3. Statistical models

As the goals of this study are correlational – to evaluate the potential existence of links between family-level intelligence and maternal health – our analytic approach focuses on correlational analyses. We note that we could fit structural equation models (SEM) to our data, but those models would necessarily imply directionality and causation. As this is the first study linking family-level intelligence to midlife maternal health, we postpone causal modeling for later research. Instead, we focus on evaluating (non-causal) links.

As in Wänström et al. (2023), growth curves were estimated, using multilevel modeling and the families in the NLSY, using the child PIAT scores. The below models are equivalent to model 1 in Wänström et al. (2023) except for the omission of the quadratic term of Child Age. The quadratic components were significant (and negative, showing a slowing increasing trend in scores) in Wänström et al. (2023), however they were small. For our current analyses, we therefore focused on the average levels (intercepts) and the average linear growths (linear slopes) per family and therefore did not include a quadratic component. Note also that our reason for fitting the below models in our current study is to save the family residuals for use in later analyses. Statistical analyses were conducted in SAS (SAS Institute Inc, 2013) version 9.4 using the procedures MIXED and CANCORR.

A family growth curve consisted of repeated measurements across all administrations for a given child, for all children in the family. Each NLSYC child had up to five repeated measurements for PIAT math, reading recognition, and reading comprehension. Multilevel models with repeated measurements at the first level, children at the second level, and mothers at the third level were estimated. We estimated models separately for the three PIAT measures, instead of adding them together as total scores, to detect differential effects. The model we estimated is the following:

(1) $${\mathrm{PiatScore}}_{tij}=\alpha_{ij}+\beta_{ij}{\mathrm{Child}}_{Age}+\epsilon_{tij},\alpha_{ij}=\alpha_{j}+v_{\alpha ij},\beta_{ij}=\beta_{j}+v_{\beta ij},\alpha_{j}\;=\alpha +u_{aj},\beta_{j}=\beta +u_{\beta j}$$

where *PiatScore_tij_* is the PIAT math-, reading recognition- or reading comprehension score, at the t:th age (with age centered around its grand mean and measured in monthly units) for the i:th child of the j:th mother, *α_ij_* is the intercept of the growth curve for the i:th child of the j:th mother, *β_ij_* is the slope of the growth curve of the i:th child of the j:th mother, *ϵ_tij_* is a residual, *α_j_* is the intercept for the j:th mother, *β_j_* is the slope for the j:th mother, *v_αij_* and *v_βij_* are child residuals, *α* is an overall intercept, *β* is an overall slope, and *u^αj^* and *u^βj^* are mother residuals. The residuals are assumed to be multivariate normally distributed within levels, and covariances between levels are assumed to be zero.

From the above estimations, we obtained growth curves, consisting of a general family-level intercept (the mean PIAT score at the average age of all children: 9.75 years) and a general slope (average monthly increase in scores), with family residuals, i.e. differences between each family’s intercept and the general intercept, and differences between each family’s slope and the general slope. These family residuals (*u_αj_* and *u_βj_*) are, except for a constant, estimates of the families’ children’s PIAT intercepts and slopes, and they were saved for use in subsequent analyses.

Lacking extensive longitudinal measures of maternal health, we evaluated difference scores for the CESD and SF-12 (physical component) variables between ages 40 and 50. Intercepts were created as the mean values of the age 40 and age 50 scores, and slopes were created as the difference between the age 50 score and the age 40 score. A positive slope (for CESD and/or SF-12) thus indicates an increase in the variable between age 40 and age 50, whereas a negative slope indicates a decrease. To examine the association between family intelligence and maternal health in middle-aged adulthood, we then correlated the family intelligence components with the maternal health components. As measures of family intelligence, we used the family (PIAT math, PIAT reading recognition, and PIAT reading comprehension) intercepts and slopes (i.e. their residuals: *u_αj_* and *u_βj_*) saved from the previous analyses. As measures of maternal health, we used the CESD and SF-12 intercepts and slopes created as described above. Because we did not assume any causal direction, and because we had multiple measures of each group of variables, we used canonical correlation analysis to estimate the correlations between composite measures of the two groups (family intelligence and maternal health).

In our final analytical step, we added maternal intelligence to the family intelligence components in the canonical correlation analyses. This addition may be viewed in two ways. One way is to view maternal intelligence as part of the estimated family intelligence (in which the composite variable is consisting of family levels and slopes of PIAT scores as well as mother intelligence levels). Another way is to view it as a statistical adjustment in our canonical correlation analyses, such that we are creating a linear equating of all families on maternal intelligence. This may be important because a possible confound in our canonical correlation analysis relating family intelligence to maternal health is maternal intelligence. Family intelligence and maternal health may be correlated simply because maternal intelligence and child intelligence are correlated, and because maternal intelligence and maternal health are correlated. This methodological adjustment is thus crucial as it addresses potential biases arising from the inherent correlations between maternal intelligence and child intelligence. Specifically, if the canonical coefficients significantly diminish after this adjustment, it suggests that previous associations might have been confounded by the interrelated nature of these variables.

## 3. Results

We begin by presenting the multilevel modeling results, utilizing the three PIAT measures. These models serve as the foundation for defining family-level IQ scores. We then present the maternal health measurements. Finally, these preliminary steps allow us to investigate the link between family-level IQ and maternal health outcomes.

### 3.1. Family intelligence

Table 1 shows results from multilevel analyses with PIAT math, reading recognition, and reading comprehension scores, as the response variable, as estimated from Eq. 1 (note that these results are almost the same results as for model 1 in Wänström et al. (2023), with the difference that a quadratic component was included in that study, as described previously). As shown, the estimated PIAT-math score at the average age (9.75 years old) is 38.9, and the estimated within-child linear increase per year is 5.2 (0.431*12 months). There is considerable variation in the intercepts of the growth curves between families ($\sigma_{u_{\alpha j}}^{2}=32.7$), but also between the children within families ($\sigma_{v_{\alpha ij}}^{2}=32.7$). Approximately 32 % of the variance in PIAT math scores is thus between-family variance (32.7/(32.7 + 22.0 + 48.7)), and approximately 21 % is between children within families (22.0/(32.7 + 22.0 + 48.7)), at the average ages of the children.

The estimated PIAT reading recognition score at the average child age is 42.5 with an average increase within child of 5.6 per year (0.469*12 months); approximately 35 % of the variance in scores is between families, and approximately 32 % is between children within families. The corresponding results for PIAT reading comprehension is an average score of 38.4 with an average within-child yearly increase of 4.6 (0.386*12 months); 31 % of the variance is between families, and 22 % is within families. As noted above, the primary purpose of this first set of analyses was to create family-level intelligence scores (residuals) to use in the correlation analyses with maternal health in sections 3.3 and following.

### 3.2. Maternal health

The mothers’ CESD scores increased between ages 40 and 50, while their physical component SF-12 scores decreased, i.e. their depressive symptoms scores increased whereas their physical health scores decreased over time; CESD_40_: *M* = 3.88, *SD* = 4.47; SF-12_40_: *M* = 5152, *SD* = 848.95; CESD_50_: *M* = 4.44, *SD* = 4.70; SF-12_50_: *M* = 4827, *SD* = 1094. Thus, their average CESD score was 4.16 with an average increase of 0.06 per year (an increase of 0.13 age 40 standard deviations across the 10-year period). Their average SF-12 score was 4990 with an average decrease of 32.5 per year (a decrease of 0.38 age 40 standard deviations across the 10-year period). The average and difference CESD and physical health scores per mother will be used to link family-level IQ (measured in Section 3.1) to maternal health. The results are reported in bivariate and canonical correlation analyses in the next sections.

### 3.3. Bivariate correlations between family intelligence and maternal health

Table 2 shows bivariate correlations between the family intelligence components and the maternal health components. As shown, the PIAT score intercepts and slopes correlate positively within subtests (0.60, 0.68, 0.61 for math, reading recognition, and reading comprehension respectively). These patterns suggest that families with high average PIAT scores (intercepts) also had steeper developments (slopes), on average. PIAT scores also correlated positively across subtests, with correlations between PIAT intercepts ranging between 0.78 and 0.89. Correlations between PIAT slopes were lower, but still fairly large (between 0.56 and 0.69). Families with higher scores, and steeper increases across ages on one PIAT subtest, thus also tended to have higher scores and increases on other subtests. The correlations are lower within the maternal health components. There is a small positive correlation (*r* = 0.05) between the CESD intercepts and slopes, indicating a weak pattern such that mothers with higher depression scores, on average, tend to have slightly larger increases in depression over time. The correlation between SF-12 intercepts and slopes is however positive and larger (*r* = 0.28), indicating that mothers with lower physical health scores decreased even more over time. The correlations between the two health measure intercepts is negative (*r* = −0.42), as expected, indicating that mothers with higher depressive scores tended to have lower physical health scores. The negative correlation (*r* = −0.15) between the CESD slope and the SF-12 slope indicates that mothers who increased in their depressive scores over time tended to decrease in their physical health scores.

The correlations across the two domains, between the family intelligence components and the maternal health components, show larger correlations between intercepts than between slopes in general. The PIAT intercepts correlate negatively with the CESD intercepts (around −0.18) and positively with the SF-12 intercepts (around 0.20). There are no significant correlations between the PIAT slopes and the maternal health component slopes. The PIAT intercepts however correlate positively with the SF-12 slopes (around 0.08), indicating that mothers with children with higher on average PIAT scores tended to not decrease as much in physical health over time.

Maternal intelligence scores are positively correlated with the PIAT intercepts (around 0.55) and slopes (around 0.40) indicating that mothers with higher intelligence when they were young tended to have families in which the children had higher average PIAT levels and steeper growth. The scores are also negatively correlated with the CESD intercepts (*r* = −0.18) and positively correlated with the SF-12 intercepts (*r* = 0.19) indicating that mothers with higher intelligence when they were young, tended to have better physical health and fewer depression symptoms when they were older. They also tended to have a less negative physical development (*r* = 0.07) and a higher increase in depression scores (although the correlation is low, *r* = 0.04).

### 3.4. Canonical correlation results

To examine the overall association between family intelligence and maternal health, we analyzed the components through canonical correlation analysis. Canonical correlations are measures that relate one composite variable to another composite variable. In the first round of canonical correlation analyses, we did not include maternal IQ in the family intelligence variable, thus relating the family intelligence components (PIAT-math, PIAT-reading recognition, and PIAT-reading comprehension intercepts and slopes) with the maternal health components (CESD and SF-12 intercepts and slopes). The first two canonical correlations are significant and are thus presented here (note that when defining canonical correlations between our two sets of variables with six components in one set, and four components in the other set, there are four canonical correlations that can be defined).

The first canonical correlation between the first composite variable of the family intelligence components and the first composite variable of the health components is 0.25 (*F*(24, 10788) = 9.81, *p* < .0001), and the second canonical correlation is 0.09 (*F*(15, 8538.8) = 1.85, *p* = .0237). The relationships (with standardized coefficients) between the canonical pairs can be expressed as follows (for explanations of abbreviations, see *Note* under Table 2):

(2) $$\begin{array}{rr} .64 & •M.In+.07•M.Sl+.45•RR.In+.00•RR.Sl-.08•RC.In+.01\\ & •RC.Sl\\ = & -.53•CESD.In+.10•CESD.Sl+.63•SF12.In+.08•SF12.Sl \end{array}$$

(3) $$\begin{array}{l} -1.12•M.In+.39•M.Sl+1.66•RR.In+.18•RR.Sl-1.13\\ •RC.In+.38•RC.Sl\\ \text{=}.03•CESD.In-.07•CESD.Sl+.43•SF\text{12}.In-1.04•SF\text{12}.Sl \end{array}$$

60 % of the variance in the family intelligence intercepts and slopes is explained by their first canonical variable (left side of Eq. 2), and 9 % of their variance is explained by their second canonical variable (left side of Eq. 3). 38 % of the variance in the maternal health intercepts and slopes is explained by their first canonical variable (right side of Eq. 2), and 21 % of their variance is explained by their second canonical variable (right side of Eq. 3). Because the magnitudes of the standardized coefficients in Eqs. 2 and 3 are comparable across components, we can see that the first composite family intelligence variable (left side of Eq. 2) is highly defined by the PIAT-math intercept and the PIAT-reading recognition intercept, with the math intercept carrying most weight. Similarly, the first composite maternal health variable (right side of Eq. 2) is defined by the CESD- and the SF12-intercepts. This first canonical correlation is attending to the intercept (level) variables. Families with high average PIAT-math scores and high average PIAT-reading recognition scores are thus linked to low maternal mental health (depression) and high maternal physical health.

The second canonical correlation is lower in magnitude, but it shows a tendency that is worth mentioning. The second family intelligence composite variable (left side of Eq. 3) is mostly defined by the difference between the PIAT-reading recognition scores, on the one hand, and the PIAT-math scores and PIAT-reading comprehension scores, on the other hand. Families with high scores on the PIAT-reading recognition subtest and low scores on the PIAT-math and the PIAT-reading comprehension subtests will have high scores on this variable. The second maternal health composite variable (right side of Eq. 3) is mostly defined by the SF12 slope, and to a lesser extent by the SF-12 intercept. Mothers with larger decreases on the SF-12 component will have higher values on this canonical variable. The small positive correlation thus indicates that mothers with children with low math and reading comprehension scores, in comparison to reading recognition scores, tend to decrease more in physical health scores.

The coefficients in Eqs. 2 and 3 above may be interpreted similarly to standardized regression coefficients, i.e. the average increase in one canonical variable when the respective variable increases by one unit (standard deviation in our case) and the other variables are constant. Even though a canonical variable may be defined by the variables with the largest absolute coefficients (as we have done above), it is still valuable to examine the correlations between the other variables and the canonical variable. Table 3 shows the correlations between each respective component and their own canonical variable.

As shown, all PIAT-math intercepts are highly positively correlated with the first canonical composite variable, as are the slopes, although to a slightly lesser extent. Although the PIAT-math and the PIAT-reading recognition subtests mostly define the first canonical variable of family intelligence, we can see that scores on all PIAT subtests, as well as subtest developments, are positively related to this variable. In contrast, the second composite family intelligence variable was defined by the difference between the PIAT-reading recognition intercepts and the PIAT-math and the PIAT-reading comprehension intercepts. Examining the correlations in Table 3, we can however see that the highest correlation is between the PIAT-reading recognition slope and the second canonical variable. Although this canonical variable was defined by the difference between the PIAT-reading recognition scores and the PIAT-math and PIAT-reading comprehension scores, this canonical variable was thus still moderately correlated with increases in PIAT-reading recognition. It should be kept in mind, however, that the coefficients in Table 3 are bivariate, and not partial, correlations between the respective component and its canonical variable (as opposed to the coefficients in Eqs. 2 and 3, which are partial coefficients). Table 3 also shows that the CESD and the SF-12 intercepts are most highly correlated with the first maternal health canonical variable, which corresponds with the previous interpretations. In addition, the SF-12 slope is highly negatively correlated with the second maternal health variable, which also corresponds with the previous interpretations.

### 3.5. Canonical correlation results - maternal intelligence

In the second round of canonical correlation analyses we included the maternal AFQT scores as a family intelligence component in the canonical correlation analyses. A new canonical correlation analysis yielded the following relationships:

(4) .40•M.In+.07•M.Sl+.43•RR.In−.02•RR.Sl−.13•RC.In−.01•RC.Sl+.43AFQT=−.51•CESD.In+.15•CESD.Sl+.64•SF12.In+.06•SF12.Sl

(5) $$\begin{array}{l} -1.18•M.In+.33•M.Sl+1.63•RR.In+.18•RR.Sl-1.14\\ •RC.In+.43•RC.Sl+.09AFQT\\ =-.02•CESD.In+.01•CESD.Sl+.35•SF12.In-1.04•SF12.Sl \end{array}$$

A comparison of Eq. 2 and Eq. 4 shows that the coefficient for the PIAT-math intercepts changed from 0.64 to 0.40, whereas the other coefficients changed very little. The family intelligence composite variable (left side of Eq. 4) may now be defined by PIAT-math and PIAT-reading recognition intercepts, as well as by maternal IQ scores. Comparisons of Eqs. 3 and 5 shows that the coefficients stayed approximately the same. The first and second canonical correlations are: 0.27 (*F*(28, 10768) = 9.46, *p* < .0001), and 0.08 (*F*(18, 8449) = 1.85, *p* = .0274) respectively. All in all, adding maternal IQ scores thus increased the first canonical correlation slightly, and decreased the PIAT-math coefficient somewhat. These results may be interpreted such that mothers with higher IQ scores as young, and with children with higher PIAT-math and PIAT-reading recognition score levels, also tend to have lower depression scores and higher physical health scores. Because adding maternal IQ did not change the interpretations from the first round of canonical correlation analyses substantially, other than increasing the correlation slightly and decreasing the PIAT math coefficient somewhat, these results also indicate that, although maternal intelligence is related to maternal health and child intelligence, it does not explain away the previously found relationships between family intelligence and maternal health, indicating that the relationship is not fully confounded by maternal intelligence.

## 4. Discussion

The aim of the current study was to examine the relationship between family intelligence and maternal mental and physical health. The bivariate correlation analyses showed that family intelligence levels, as well as maternal IQ, were positively correlated with maternal physical health levels and negatively correlated with maternal depressive symptoms levels. These findings are consistent with earlier findings showing direct links between maternal intelligence and later maternal health, as well as child intelligence and maternal health. In particular, we found that mothers in families strong in math, reading recognition and reading comprehension had fewer depression symptoms and more positive physical health. Family intelligence levels also correlated with maternal physical health change, and mothers in those families also tended to show less decline in physical health over time.

When examining these associations simultaneously through canonical correlation analyses, the first canonical correlation between the composite of family intelligence and the composite of maternal health was *R* = 0.25, suggesting a moderate and meaningful relationship between family intelligence and maternal health. The second canonical correlation (by definition orthogonal to the first) was smaller, *R* = 0.09, but also statistically significant. Results indicated that math and reading recognition levels, in particular, showed strong links to maternal health, and that low scores on math and reading comprehension tended to be associated with steeper declines in physical health. Several other components however also correlated (bivariately) with the canonical composite variables, indicating that mothers in families strong in all three PIAT test areas as well as in growth experienced more positive physical health and less depressive symptoms, and mothers in families with strong PIAT reading recognition growth experienced less decline in physical growth.

When maternal intelligence was added as part of the family intelligence (together with children’s intelligence levels and growths) it strengthened the relationship between family intelligence and maternal health somewhat, which might indicate that family intelligence is measured more reliably using both child- and mother-scores. In addition, the links between the child components and the maternal health components did not disappear when maternal intelligence was added to the canonical family intelligence variable, although maternal intelligence in early life, in itself, was associated with maternal health later in life. This suggests that it was meaningful to include both components of the children and of the mothers into the family intelligence composite variable, and that maternal intelligence did not account for enough variance in maternal health to decrease the coefficients for the child components substantially.

### 4.1. Interpretations

Our findings may have several interpretations. Family intelligence may influence maternal health (although we are cautious in making causal statements, because we did not predict a causal direction). Children with lower cognitive performance, and children with mental delays, may for example have more behavioral problems (as reported by the mother, Caplan et al., 1989), affecting maternal mental and physical health negatively (Baker et al., 1997; Caplan et al., 1989; Eisenhower et al., 2009; Soares et al., 2024). Alternatively, or in addition, maternal health may influence family intelligence. Mothers who have better health are better equipped to help their children in their upbringing and general cognitive development, with schoolwork etc. Maternal depression has been found to be negatively related to children’s cognitive functioning (Sutherland et al., 2021) and overall health (Hardie & Landale, 2013). Mothers with depressive symptoms may spend less time with their children (e.g. Frech et al., 2011) and are at greater risk of parenting challenges (such as harsh parenting; Guyon-Harris et al., 2022). Children of mothers with poor health who also lack economic and other resources may also be at greater risk for poor health (e.g. Hardie & Landale, 2013). The relationship between maternal health and child intelligence may also be reciprocal. Garbarski (2014) studied children and mothers in the NLSY and found both that child activity limitations affected maternal health and that maternal health affected activity limitations. Future studies may look more closely at the causal directionality of the associations found in our study, for example by estimating paths in SEM models, or alternatively, by designing instrumental variable studies. An example of such a directionality model is a mediation model, in which family intelligence (composed of child components) mediates the relationship between maternal intelligence and maternal health. This model can be fit with our results, but because we did not predict this directional relationship, we save such an analysis for future confirmatory research.

### 4.2. The Flynn effect and the relationship between family intelligence and health

Wänström et al. (2023) found that later-born family cohorts (later-born because the mother was born later or because the first child was born later) tended to have higher levels and bigger increases in family intelligence (family Flynn effects). In the current study we found that mothers in higher intelligence families tended to have better mental and physical health at middle-age. This finding – which might be characterized as a “Flynn effect transfer” – shows that intellectual levels and growth within the family has broad implications. Higher family intelligence is certainly meaningful and important in its own right. As parents and children within families constantly interact, it is expected that both levels and increases in the cognitive performances of the children in families may have impacts on the entire family. For example, Hadd and Rodgers (2017) used the NLSY and showed that children contributed substantially to constructing the cognitive components of their family environment. Further, O’Keefe and Rodgers (2022) showed a type of Flynn effect associated with the cognitive family environment itself, as that component showed cohort increases over time. However, as later family cohorts achieve higher intellectual performance over time (family Flynn effects) there may also be downstream maternal health effects that are correlated with those family Flynn effects. The longitudinal data structure in the NLSY enabled the construction of slopes and intercepts for maternal health, assessing both physical and mental health changes between ages 40 and 50 for the NLSY79 cohort, thus enabling us to investigate the relationship between family intelligence and maternal health levels and slopes. Other downstream effects may be identified and evaluated in future research (for both mothers and children).

The Parental Executive Model (Rodgers & O’Keefe, 2023) and the Life-History Perspective (Woodley, 2012) lead to similar predictions. At the family level, the Life History Perspective suggests that families oriented toward a slow life-history strategy (i.e., families oriented toward a K reproductive strategy that focuses on fewer children and greater parental investment) invest planning, fertility intentionality, and focus on health outcomes among their children. Within the Parental Executive Model, these actions are specifically oriented toward parental attention to supporting and actively influencing childhood intellectual development. In each case, it makes theoretical sense that parents oriented toward positive childhood outcomes such as intellectual development would also ultimately pay particular attention to their own health outcomes as well. Both models would predict delays in the start of a family, which links them explicitly to family Flynn effects, and positive relationships between family intelligence and maternal mental and physical health. The theoretical predictions that emerge from these theories and the current results are testable empirically, and would motivate future research more specifically tied to either or both of these theoretical orientations.

As mentioned previously, we did not assume any causal directions between family intelligence and maternal health in our correlational analyses. A possible Flynn effect transfer however suggests that increases in family intelligence across cohorts may have beneficial effects on later maternal health across cohorts. Recent Flynn effect research has begun exploring the effect and its implications in older age groups. Studying older age groups however comes with certain difficulties. A notable distinction in studying older demographics is the necessity to consider selection bias introduced by mortality rates, which are negligible during childhood. Moreover, the factors influencing cognitive impairments, such as dementia, in older adults differ significantly from those affecting intellectual development in children. Clouston et al. (2021) advocated for studies that connect the Flynn effect with cognitive decline in older adults, especially in light of findings that suggest less pronounced cognitive decline in more recent cohorts (Dodge et al., 2017; Elwood et al., 2013). Dickinson and Hiscock (2010) highlighted this aspect by comparing WAIS scores between individuals aged 20 and those aged 70, initially observing a significant cognitive decline with age. However, when adjusting for the Flynn effect, they found that only 15 % of the decline was attributable to within-individual cognitive declines (i.e., patterns that could not be attributed to generational differences in intelligence), whereas 85 % was related to the Flynn effect itself. Further research into the Flynn effect concerning older adults has documented variations over time. Unlike in younger populations, these changes are attributed to different factors, often showcasing a decrease in dementia levels across generations. Significant contributions in this area include studies by Hessel et al. (2018), O’Keefe et al. (2024), Rocca et al. (2011), Skirbekk et al. (2013), and Zhang et al. (2024), each exploring distinct aspects of cognitive trends in aging populations and their potential links to the Flynn effect. Despite the difficulties in studying aging populations, future studies linking family intellectual development (in childhood) to health and cognitive trajectories in older ages would contribute to shed light on our results.

As mentioned previously, causal directions may however also operate in the opposite direction. Better maternal mental and physical health may have positive effects on family cognitive performance and growth. Viewed in this way, improved maternal health across cohorts may instead (or in addition) be seen as part of an explanation to increasing family intelligence scores over time (family Flynn effects), and is consistent with the inter-generational components of the Parental Executive Model. Future studies may focus on studying both cohort changes in family intelligence and cohort changes in health measures to investigate similarities in patterns over time.

### 4.3. Strengths and limitations

In the current research, we have employed a novel methodological approach by conceptualizing intelligence at the family level, and extracting family measures from multilevel growth models - intercepts and slopes – enabling us to examine links between family intelligence and maternal health levels and change. Our research was motivated by frameworks proposed by O’Keefe and Rodgers (2017) and Wänström et al. (2023), in which family variance was a dominant explanatory component of cohort changes in intelligence. Our analysis predominantly relied on correlational methods due to the scant existing literature to inform our hypotheses. However, earlier studies that established the presence of a Flynn effect in the NLSY family data (e.g., Ang et al., 2010; Rodgers & Wänström, 2007; Wänström et al., 2023) provided direction for our measurement and design strategies. Therefore, our study aligns more with what Fife and Rodgers (2022) term a “rough CDA” study within the exploratory-confirmatory research spectrum, implying that while it is informed by previous research and theories, it does not extend to making definitive confirmatory predictions. We anticipate that the findings from this study will lay the groundwork for future research that can further explore empirical and theoretical dimensions based on these initial results.

It is a challenge to find data sources that can be used to do the types of analyses that we present here, based on longitudinal family-level data. Besides the NLSY – which is an excellent source for this purpose – a few others exist. It would substantially improve the scientific contribution of the current work for replications – ideally conceptual replications that would expand the current work in important ways – by different research teams using different data sources from other populations.

A limitation of our study is the narrower scope of maternal health measures at ages 40 and 50, compared to the more comprehensive family intelligence measures derived from PIAT scores during children’s ages of 5 to 14. Extending this research into longitudinal data encompassing older ages would constitute a significant next step.

The NLSY data contain health information on the mothers, and not the fathers, and we were therefore only able to use information on the mothers. Maternal health is obviously an important outcome, because of its link to child development (nutrition during pregnancy, time spent with children etc.) as well as being influenced by the family (e.g. by family stressors). Our correlational analyses indicated that the family intelligence growth curve levels and increases were related to maternal physical and mental health, at ages 40 and 50, and (to a lesser extent) to health changes between ages 40 and 50. Future research using other data sources may investigate links to paternal health as well.

Future studies that leverage predictions out of this (and other) research can obviously extend those predictions into more formal confirmatory analyses. The type of mediation study – using, for example, family intelligence as a mediator of the link between maternal intelligence and maternal midlife health – would be such a study. Others exist as well, of course.

### 4.4. Summary

Our study evaluated the association between family-level intelligence and maternal health outcomes in middle age, using data from the National Longitudinal Survey of Youth (NLSY79 and NLSY-Children). The investigation was motivated by established associations between individual intelligence levels and health outcomes, and by the pronounced importance of between-family variance in intelligence scores (Wänström et al., 2023), making it possible to extend this inquiry to the family context. Our results expand on previous positive links between intelligence and health, by considering the family as a unit, and by examining both levels and changes in intelligence as they link to maternal outcomes. Canonical correlation analysis identified a moderate but significant relationship between family intelligence and maternal health. The results indicated that family-level intelligence is positively related to maternal health at middle-age. We have discussed the notion of a “Flynn effect transfer” within families, such that families with higher cognitive levels and gains, possibly due to the mothers in the families belonging to later cohorts, or delayed childbirth (i.e. to later starting families), exhibit better maternal health outcomes in midlife, which could be studied in future research. Our results provide a foundation for future research to explore the downstream effects on both mothers and children, emphasizing the value of intellectual development within families for enhancing health outcomes over time and across generations.

## Figures and Tables

**Table 1:** Estimates and standard errors (S.E.) from analyses of three-level models (Eq. 1).

Variable	PIAT Math		PIAT Read Rec		PIAT Read Comp	
	Estimate	S.E.	Estimate	S.E.	Estimate	S.E.
Intercept	38.944***	0.114	42.511***	0.136	38.363***	0.114
Child age	0.431***	0.002	0.469***	0.002	0.386***	0.002
$\sigma_{u_{\alpha j}}^{2}=32.7$	32.747***	1.162	44.547***	1.640	31.813***	1.159
$\sigma_{u_{\beta j}}^{2}=32.7$	0.003***	0.0002	0.007***	0.0003	0.004***	0.0003
$\sigma_{v_{\alpha ij}}^{2}=32.7$	22.020***	0.711	41.731***	1.069	22.815***	0.739
$\sigma_{v_{\beta ij}}^{2}=32.7$	0.001***	0.0003	0.005***	0.0003	0.001***	0.0003
$\sigma_{\epsilon_{tij}}^{2}=32.7$	48.728***	0.496	42.327***	0.450	48.462***	0.512
−2ResLogLikelihood	247,534.6		249,415.3		241,893.6	

*Note*. Because the estimates of the variances of the slopes were very small, correlations between intercepts and slopes were not estimated.

*** *p* < .001,

**Table 2:** Bivariate correlations between intelligence components and maternal health components.

Component	M.In	RR.In	RC·In	M.Sl	RR.Sl	RC.Sl	CESD·In	SF12·In	CESD.Sl	SF-12.Sl	Moth.IQ
M.In	1.00	0.78***	0.80***	0.60***	0.53***	0.52***	−0.20***	0.21***	0.01	0.10***	0.59***
RR.In		1.00	0.89***	0.43***	0.68***	0.48***	−0.18***	0.21***	0.00	0.06**	0.51***
RC·In			1.00	0.46***	0.60***	0.61***	−0.17***	0.19***	0.00	0.08***	0.55***
M.Sl				1.00	0.56***	0.57***	−0.12***	0.14***	0.02	0.03	0.40***
RR.Sl					1.00	0.69***	−0.13***	0.15***	0.01	0.01	0.39***
RC.Sl						1.00	−0.11***	0.12***	0.02	0.02	0.42***
CESD·In							1.00	−0.42***	0.05**	−0.18***	−0.18***
SF12·In								1.00	−0.05*	0.28***	0.19***
CESD.Sl									1.00	−0.15***	0.04*
SF12.Sl										1.00	0.07**
Moth.IQ											1.00

* *p* < .05,

** *p* < .01,

*** *p* < .001.

*Note*. M.In = PIAT math intercept, RR.Sl = PIAT reading recognition slope, RR.In = Piat reading recognition intercept, RC.Sl = PAIT reading comprehension slope, RC·In = Piat reading comprehension intercept, RC.Sl = Piat reading comprehension slope, CESD·In = CESD intercept, CESD.Sl = CESD slope, SF-12.In = SF12 intercept, SF12.Sl = SF-12 slope, Moth.IQ = AFQT score.

**Table 3:** Correlations between components and their canonical variables.

Family intelligence components	Family intelligence 1	Family intelligence 2	Maternal health components	Maternal health 1	Maternal health 2
M.in	0.97	−0.22	CESD.in	−80	0.03
M.sl	0.61	0.25	CESD.sl	0.03	0.07
RR.in	0.91	0.26	SF12.in	0.87	0.13
RR.sl	0.64	0.53	SF12.sl	0.33	−0.91
RC.in	0.87	−0.04			
RC.sl	0.54	0.26			

*Note*. M.In = PIAT math intercept, RR.Sl = PIAT reading recognition slope, RR.In = Piat reading recognition intercept, RC.Sl = PAIT reading comprehension slope, RC·In = Piat reading comprehension intercept, RC.Sl = Piat reading comprehension slope, CESD·In = CESD intercept, CESD.Sl = CESD slope, SF-12.In = SF12 intercept, SF12.Sl = SF-12 slope.

## Data Availability

We use a public data source (available here: https://www.nlsinfo.org/ )
